# The Role of Beta-Defensin 2 in Preventing Preterm Birth with Chorioamnionitis: Insights into Inflammatory Responses and Epithelial Barrier Protection

**DOI:** 10.3390/ijms26052127

**Published:** 2025-02-27

**Authors:** Sangho Yun, Shin-Hae Kang, Jiwon Ryu, Kyoungseon Kim, Keun-Young Lee, Jae Jun Lee, Ji Young Hong, Ga-Hyun Son

**Affiliations:** 1 Institute of New Frontier Research Team, College of Medicine, Hallym University, Chuncheon 24252, Republic of Korea; z4213@daum.net (S.Y.); shinhae0808@naver.com (S.-H.K.); iloveu59@hallym.or.kr (J.J.L.); 2 Division of Maternal-Fetal Medicine, Department of Obstetrics and Gynecology, Hallym University College of Medicine, Kangnam Sacred Heart Hospital, Seoul 07441, Republic of Korea; jwryu@hallym.or.kr (J.R.); kristy_kks@naver.com (K.K.); mfmlee@hallym.ac.kr (K.-Y.L.); 3 Departments of Anesthesiology and Pain Medicine, Hallym University College of Medicine, Chuncheon 24252, Republic of Korea; 4 Division of Pulmonary, Allergy and Critical Care Medicine, Department of Internal Medicine, Sacred Heart Hospital, Hallym University Medical Center, Chuncheon 24253, Republic of Korea

**Keywords:** antimicrobial peptides, beta-defensin, chorioamnionitis, preterm birth

## Abstract

Antimicrobial peptides, such as beta-defensin 2 (BD2), are vital in controlling infections and immune responses. In this study, we investigated the expression and role of BD2 in the amniotic membrane and human amniotic epithelial cells (hAECs) from patients with preterm birth and chorioamnionitis, focusing on its regulation of inflammatory cytokines and its protective effect on the epithelial barrier. Our results show increased BD2 expression in chorioamnionitis, and Lipopolysaccharide (LPS)-induced inflammation increased BD2 release from hAECs in a dose- and time-dependent manner. BD2 treatment effectively modulated the inflammatory response by reducing pro-inflammatory cytokines (IL-6, IL-1β) and enhancing the release of the anti-inflammatory cytokine IL-10. Additionally, BD2 helps preserve epithelial barrier integrity by restoring E-cadherin expression and reducing Snail expression in inflamed hAECs. In an LPS-induced preterm birth mouse model, BD2 treatment delayed preterm delivery and reduced inflammatory cytokine levels. These results suggest that BD2 plays a protective role in preventing preterm birth by regulating inflammation and maintaining epithelial barrier function, highlighting its therapeutic potential for inflammation-related preterm birth.

## 1. Introduction

Antimicrobial peptides (AMPs), also known as cationic host defense peptides, are a class of small peptides capable of controlling infections through their antimicrobial properties and modulating hosts’ immune responses [1,2,3,4]. AMPs are expressed in most parts of the human body exposed to microbial environments, including the skin, intestinal mucosa, lungs, oral mucosa, eyes, and reproductive tract [2,4]. While some AMPs are consistently expressed, the majority are induced in response to an infection, inflammation, or injury [1,2]. Dysregulation of AMP expression can compromise antimicrobial defense and has been associated with the pathogenesis of infectious and inflammatory diseases, including Crohn’s disease and atopic dermatitis [5,6,7,8,9,10].

Preterm birth (PTB), defined as delivery before 37 weeks of gestation, is the most frequent cause of infant mortality, accounting for at least one-third of all infant deaths. Neonates born preterm are at increased risks of long-term morbidity, including neurodevelopmental impairments, as well as respiratory and gastrointestinal complications [11,12,13]. While multiple pathological mechanisms contribute to preterm labor, such as uterine overdistension, uteroplacental hemorrhage, and various immunologically mediated processes, ascending infection of vaginal microbiota is recognized as a major contributor to spontaneous PTB (sPTB) [11,13,14]. During pregnancy, AMPs are expressed in the cervix, vagina, uterine wall, amniotic fluid, fetal membranes, placenta, and fetus, where they play a crucial role in protecting against intrauterine infections and inflammatory stimuli [15,16,17,18,19,20,21,22,23,24,25]. In particular, clinical conditions associated with sPTB, such as preterm labor, preterm premature rupture of membranes (PPROM), and cervical insufficiency, have been linked to altered AMP expression in the cervicovaginal secretions and amniotic fluid [23,24,26,27,28].

Among the major AMPs, defensins are characterized by a conserved β-sheet core stabilized by three disulfide bonds formed between six cysteine residues [29,30]. Based on the configuration of these disulfide bonds, defensins are classified into three subgroups: α-, β-, and θ-defensins [2,29]. Human α-defensins are primarily produced by neutrophils and Paneth cells of the small intestine [30,31,32,33]. In contrast, human β-defensins (BDs) are mainly expressed in epithelial cells, providing a protective function in microbial-exposed sites such as the respiratory, intestinal, and genitourinary tracts, as well as the skin [31]. Human BD1–3s have been identified in the placenta, chorionic trophoblasts, amniotic epithelium, and decidua, where they act as crucial components of mucosal innate immunity [16,23,34,35,36].

Recent studies reported elevated β-defensin levels in amniotic fluid in sPTB-related conditions, including intra-amniotic infection, inflammation, PPROM, and cervical insufficiency [23,24,26,27,28,37]. While previous studies primarily focused on BD2 elevation in these conditions, recent findings suggest that higher vaginal BD2 levels during mid-pregnancy are associated with a reduced risk of sPTB [27]. These findings indicate that although BD2 levels rise in response to inflammation in sPTB, its baseline levels during mid-pregnancy may influence pregnancy outcomes and play a protective role in preventing PTB. Furthermore, AMPs, including BD2, can be secreted from the amniotic membrane in response to inflammatory cytokines such as IL-1β [25,38]. This result suggests that the antibacterial activity of the amnion and the presence of AMPs in the amniotic fluid serve as mechanisms to protect the fetus from infection.

While previous research examined changes in AMP expression and their association with sPTB-related conditions, the specific role of β-defensin in sPTB-associated inflammation remains unclear. Therefore, this study aims to investigate BD2 expression in the amniotic membrane of patients with sPTB and chorioamnionitis. Moreover, we intend to reveal BD2 functions in intrauterine infection and inflammation associated with sPTB. Finally, we assessed whether BD2 effectively prevents preterm birth in a mouse model of intrauterine inflammation-induced preterm birth.

## 2. Results

### 2.1. Beta-Defensin 2 Expression in the Amniotic Membrane Is Increased in Preterm Birth with Chorioamnionitis

**Figure 1:**
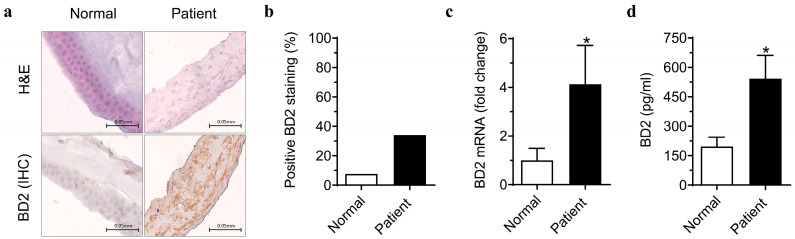
Beta-defensin 2 expression in amniotic membrane. Paraffin-embedded sections from the normal and patient group were stained with H&E and BD2-specific immunohistochemistry (**a**). Quantitative comparison of BD2 staining intensity using ImageJ software (**b**). Expression levels of BD2 mRNA and protein release from hAECs from normal and patient groups (**c**,**d**). * Significant difference (*p* < 0.05) compared with normal group. BD2, β-defensin 2; hAECs, human amniotic epithelial cells.

After fixing and sectioning the amniotic membrane from the normal group (*n* = 3) and preterm patient diagnosed with chorioamnionitis (*n* = 3) in paraffin, we analyzed BD2 expression using immunohistochemistry. The BD2 expression was predominantly localized to the epithelial and sub-epithelial stromal layer of the amniotic membrane. In comparison to the normal controls, the patient group exhibited more brown-stained areas. Analysis using the ImageJ software (ver. 1.53e, National Institutes of Health, Bethesda, MD, USA; https://imagej.nih.gov/ij/, accessed on 3 November 2023) revealed a higher proportion of BD2-stained areas in the amniotic membrane of preterm patients with chorioamnionitis compared to controls (Figure 1a,b).

Moreover, the mRNA expression of BD2 in human amniotic epithelial cells (hAECs) isolated from the patient group was higher than that in the control group. Furthermore, the BD2 levels in the culture medium of hAECs were significantly elevated in the patient group. These findings indicate an increased expression of BD2 in the amniotic membrane and hAECs of patients who experienced preterm delivery with chorioamnionitis, as well as an increase in BD release from hAECs (Figure 1c,d).

**Figure 2:**
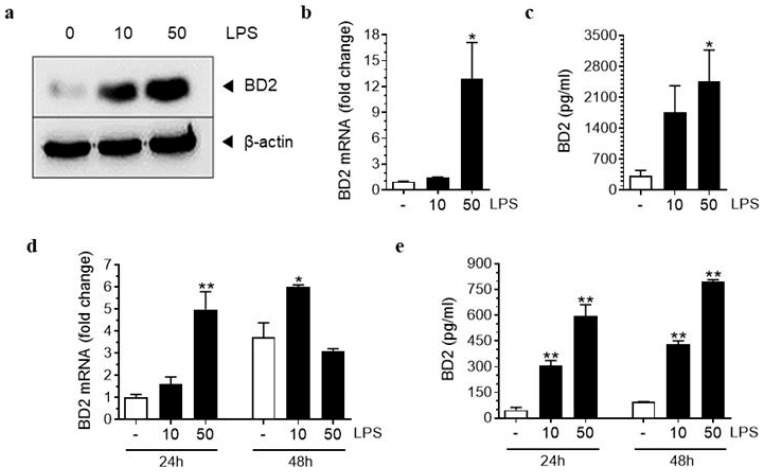
Beta-defensin 2 expression in human amniotic epithelial cells. BD2 protein and mRNA expression in hAECs treated with LPS at concentrations of 10, and 50 ng/mL for 24 h (**a**–**c**). Comparison of BD2 mRNA expression levels and protein release from hAECs in response to varying LPS doses and administration durations (**d**,**e**). * Significant difference (*p* < 0.05) compared with normal group. ** Significant difference (*p* < 0.01) compared with normal group. BD2, β-defensin 2; hAECs, human amniotic epithelial cells; and LPS, lipopolysaccharide.

To determine whether the increase in BD2 in the patient group was due to inflammation, hAECs isolated from the normal group were treated with lipopolysaccharide (LPS) (10 or 50 ng/mL) to induce inflammation mimicking chorioamnionitis. BD2 mRNA and protein expression were significantly increased in LPS-treated hAECs compared to that in normal controls. In addition, a significant elevation in BD2 levels was observed in the culture medium of LPS-stimulated hAECs compared to that in the medium from normal controls (Figure 2a–c). BD2 levels in the culture medium of hAECs were significantly higher with 50 ng/mL of LPS treatment compared to 10 ng/mL. Furthermore, when comparing treatment durations, the group treated with LPS for 48 h exhibited higher BD2 levels in the culture medium than the group treated for 24 h. These findings indicate that the release of BD2 from hAECs treated with LPS is both dose- and time-dependent (Figure 2d,e).

### 2.2. Effect of Beta-Defensin 2 on LPS-Induced Inflammation in Human Amniotic Epithelial Cells

**Figure 3:**
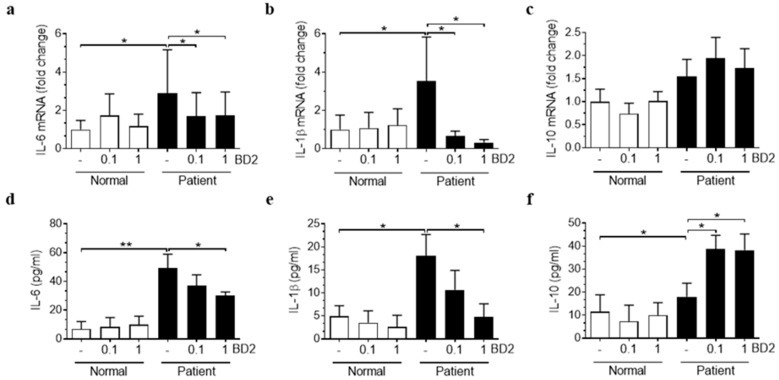
Effect of beta-defensin 2 on the expression of inflammatory cytokines in human amniotic epithelial cells. IL-6, IL-1β, and IL-10 mRNA expression in hAECs and protein levels in the culture medium of hAECs from normal and patient groups, with or without BD2 0.1 g/mL, 1.0 μg/mL co-treatment (**a**–**f**). * Significant difference (*p* < 0.05), and ** significant difference (*p* < 0.01). BD2, β-defensin 2; hAECs, human amniotic epithelial cells; and LPS, lipopolysaccharide.

To investigate the effect of BD2 on the amniotic membrane in patients with chorioamnionitis, we compared the levels of inflammatory cytokines, including IL-6 and IL-1β, and anti-inflammatory cytokines such as IL-10 between the patient and normal groups. hAECs isolated from the patient group (patient-derived hAECs, PD-hAECs) exhibited significantly higher mRNA expression levels of IL-6 and IL-1β compared to the control group, with no significant difference in IL-10 between the two groups (Figure 3a–c). Similarly, the levels of IL-6 and IL-1β in the culture medium of PD-hAECs were significantly higher than those in the culture medium of normal controls. To assess the effect of BD2 on the inflammatory response in the amniotic membrane, PD-hAECs were treated with BD2, which significantly reduced the elevated levels of IL-6 and IL-1β while increasing IL-10 levels in the culture medium (Figure 3d–f). These findings were consistent in hAECs from the normal group following LPS treatment, which significantly increased IL-6 and IL-1β expression at both the mRNA and protein levels. BD2 co-treatment mitigated this increase, while also enhancing IL-10 expression in a dose-dependent manner, with significant changes in the culture medium observed at 1 µg/mL BD2’s (Supplementary Figure S1). When examining other cytokines associated with intrauterine infection and inflammation, such as IL-8, MCP-1, MMP9, MMP8, and MMP2, their expression levels remained unchanged following BD2 co-treatment with LPS (Supplementary Figure S2).

**Figure 4:**
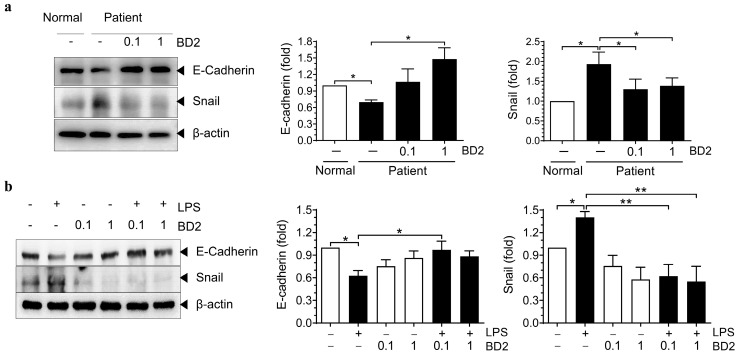
Effect of Beta-defensin 2 on cell adhesion molecules expression in human amniotic epithelial cells. E-cadherin and Snail expression in hAECs from normal and patient groups treated with 0.1 µg/mL or 1 µg/mL BD2, with quantitative comparison using ImageJ software (**a**). E-cadherin and Snail expression in hAECs from normal controls treated with 10 ng/mL LPS and 0.1 µg/mL or 1 µg/mL BD2 for 24 h, with quantitative comparison using ImageJ software (**b**). * Significant difference (*p* < 0.05), and ** significant difference (*p* < 0.01). hAECs, human amniotic epithelial cells; BD2, β-defensin 2; and LPS, lipopolysaccharide.

Chorioamnionitis can compromise the amniotic epithelial barrier, increasing its permeability to pathogens, with the accompanying elevation of proinflammatory cytokines further contributing to the barrier weakening [39,40,41,42]. Therefore, we subsequently investigated the effect of BD2 on the amniotic epithelial barrier under inflammatory conditions such as chorioamnionitis. We examined the expression of the epithelial surface marker E-cadherin and the transcription factor Snail, a direct repressor of E-cadherin, in PD-hAECs. PD-hAECs exhibited significantly lower E-cadherin expression compared to the control group. However, treatment with BD2 in PD-hAECs significantly increased E-cadherin expression. In contrast, Snail expression was significantly higher in PD-hAECs than in the control group, but BD2 treatment significantly reduced Snail expression. Similar results were observed in LPS-treated hAECs (Figure 4a). LPS treatment significantly reduced E-cadherin expression, but co-treatment with BD2 restored its expression. Additionally, Snail expression was markedly elevated in LPS-treated hAECs; however, co-treatment with BD2 and LPS significantly reduced Snail expression (Figure 4b). These findings suggest that the amniotic epithelial barrier is compromised during LPS-induced inflammation. BD2 plays a protective role by preserving E-cadherin expression and suppressing Snail expression, thereby mitigating barrier damage.

### 2.3. NF-kB Mediated Regulation of Beta-Defensin 2 Expression in hAECs During LPS-Induced Inflammation

**Figure 5:**
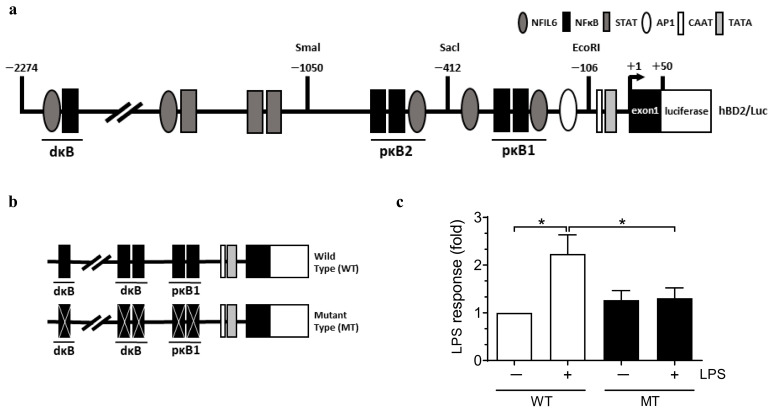
Schematic representation of the cis-element arrangement in the BD2 promoter constructs and luciferase assay for BD 2 promotor activity. Diagram of the BD2 promoter constructs used in the luciferase reporter assay. Constructs include wild-type (WT) and various deletion mutants, as indicated by shaded and open circles representing regulatory elements. hAECs were transfected with either wild-type or mutant constructs (**a**). The schematic diagram highlights the differences between wild-type and mutant constructs (**b**). Following stimulation with 100 ng/mL LPS for 6 h, promoter activity was quantified using a luciferase assay. Luciferase activity is expressed as fold change relative to the control group (**c**). * Significant difference (*p* < 0.05).

NF-kB is a central regulatory pathway induced by LPS in inflammation. BD2 has binding sites on the promoter that interact with NF-kB at positions −2193 to −2182 (dκB), −596 to −572 (pκB2), and −205 to −186 (pκB1) (Figure 5a). A vector with mutations in these binding sites was transfected into hAECs, followed by LPS treatment for 6 h. LPS treatment led to high luciferase expression in the wild-type (non-mutated) cells. However, in the mutant type with NF-kB site mutations, this expression was lower than that in the wild type (Figure 5b,c). This result indicates that BD2 expression in hAECs is regulated by NF-kB during LPS-stimulated inflammation.

### 2.4. The Effect of Beta-Defensin 2 in an LPS-Induced Preterm Birth Mouse Model

**Figure 6:**
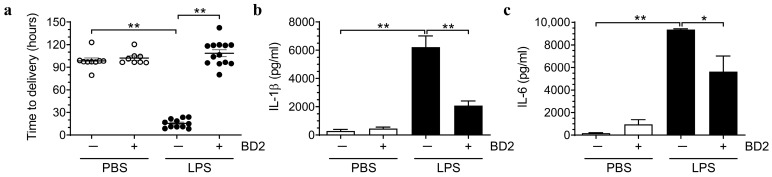
Comparison of time to delivery and inflammatory cytokines levels. Comparison of time to delivery among the PBS-treated group (100 μL, *n* = 9), BD2-only group (15 μg/100 μL, *n* = 8), LPS-only group (5 μg/100 μL, *n* = 11), and LPS + BD2 group (5 μg/100 μL and 15 μg/100 μL, respectively, *n* = 13) (**a**). Plasma inflammatory cytokine levels among the same groups (**b**,**c**). * Significant difference (*p* < 0.05), and ** Significant difference (*p* < 0.01). PBS, phosphate-buffered saline; BD2, β-defensin 2; and LPS, lipopolysaccharide. phosphate-buffered saline.

To evaluate the potential of BD2 in preventing preterm birth in vivo, we employed an LPS-induced preterm birth mouse model [43,44,45]. Four experimental groups were established based on the intrauterine treatment received: a control group treated with phosphate-buffered saline (PBS) (100 μL, *n* = 9), an LPS-treated group (5 μg/100 μL, *n* = 11), a BD2-only-treated group (15 μg/100 μL, *n* = 8), and a group treated with both LPS and BD2 (5 μg/100 μL and 15 μg/100 μL, respectively, *n* = 13). In the BD2-only-treated group and the LPS + BD2-treated group, BD2 was additionally administered subcutaneously 24 h before intrauterine BD2 injection. In the LPS-treated group, delivery occurred within 24 h after LPS administration (median (range) 17.8 h (11.0–23.1 h)). In contrast, the BD2-only and the LPS + BD2-co-treated group exhibited delivery timings comparable to the control group (median (range) 99.1 h (96.5–104.7 h), 112.3 h (98.4–119.6 h), and 97.9 h (97.5–98.9 h), respectively) with no significant differences observed between groups (Figure 6a). Therefore, BD2 treatment significantly extended the pregnancy duration compared to the LPS-treated group, where preterm birth occurred (LPS-treated group vs. LPS + BD2-co-treated group, *p* < 0.001). Moreover, we analyzed the levels of the inflammatory cytokines IL-6 and IL-1β between the groups, and the results demonstrate that BD2 significantly reduced the LPS-induced elevation of these cytokines (Figure 6b,c). These findings suggest that BD2 has therapeutic potential for preventing preterm birth by regulating inflammatory cytokines and are consistent with previous in vitro results.

## 3. Discussion

Our results demonstrate a significant increase in the expression of BD2 in the amniotic membrane of patients with chorioamnionitis, as well as in isolated hAECs from preterm patients with chorioamnionitis. LPS-induced inflammation elevates BD2 expression in hAECs in a dose- and time-dependent manner. Moreover, our study using hAECs revealed that BD2 modulates the inflammatory response and plays a role in preserving the integrity of the epithelial barrier under inflammatory conditions associated with preterm birth. Additionally, our study found that BD2 expression in hAECs is regulated by NF-kB during LPS-induced inflammation. Furthermore, we explored the therapeutic potential of BD2 in preventing preterm birth using an LPS-induced mouse model. Our findings demonstrate that BD2 effectively mitigates LPS-induced preterm delivery and reduces inflammatory cytokine levels, highlighting its potential as a therapeutic agent for inflammation-associated preterm birth.

Beta-defensins are expressed in the pregnant uterus: BD1–3 are detected in the placenta and chorion trophoblasts, amniotic epithelium, decidua, and amniotic fluid, whereas BD1 and BD2 mRNA are expressed in the chorion, villus, and placental tissues [18,20,22,46]. Previous studies demonstrated that BD2 concentration in the amniotic fluid is markedly increased in women with intra-amniotic infection/inflammation. Additionally, patients who delivered preterm had higher amniotic fluid BD2 concentrations than those who delivered at term [37,47]. Furthermore, the levels of BD2 in the amniotic fluid were higher in women with cervical insufficiency than in normal controls [24]. In addition, proteomic analysis of amniotic fluid revealed that defensins exhibit a distinctive proteomic profile associated with intrauterine inflammation and chorioamnionitis, highlighting their potential as biomarkers for the early recognition of these conditions [28,48,49,50]. As demonstrated in previous studies, defensins have been reported to be elevated in the amniotic fluid of patients exhibiting symptoms associated with sPTB. Consequently, defensins have been highlighted as markers associated with microbial invasion of the amniotic cavity, intrauterine inflammation, and chorioamnionitis. However, a recent study demonstrated an association between higher vaginal BD2 levels at mid-pregnancy and reduced risk of sPTB [27]. Moreover, high maternal stress and low cervicovaginal defensin 2 levels are associated with an increased risk of sPTB [51]. Furthermore, although BD2 levels are higher in women with cervical insufficiency compared to normal controls, elevated amniotic fluid BD2 levels at mid-pregnancy have been associated with favorable pregnancy outcomes in women with cervical insufficiency [24]. These findings indicate that the differential expression of defensins, depending on the severity of infection and the inflammatory response, can significantly influence pregnancy outcomes.

In an experimental study using amniotic epithelial cells, treatment with LPS led to a marked upregulation of BD3 mRNA expression, and the BD3 protein in the amniotic sections was intensively positive in women with histological chorioamnionitis who delivered preterm compared to control patients who delivered at term [34]. Moreover, exposure of the amniotic membrane to IL-1β leads to increased secretion of AMPs: BD-2, BD3, cathelicidin, and elafin [25,38]. Thus, amnion epithelial cells produce AMPs in the amniotic fluid in response to infection or inflammation and contribute to the innate immunity of the intra-amniotic cavity that protects the fetus during pregnancy [52,53]. Consistent with previous studies, this study demonstrated that BD2 expression is elevated in the amniotic membranes, with increased BD2 release observed in preterm patients with chorioamnionitis and in hAECs stimulated by LPS. These findings suggest that BD2 plays a crucial role in the amniotic membrane’s response to infection and inflammation, contributing to physiological defense mechanisms that support pregnancy maintenance.

To further investigate the role of BD2 in inflammatory conditions, hAECs were co-treated with BD2 during LPS exposure. This significantly reduced the expression of pro-inflammatory cytokines, such as IL-6 and IL-1β, at both the mRNA and protein levels, while increasing the expression and release of the anti-inflammatory cytokine IL-10. These findings indicate that BD2 not only suppresses excessive inflammation, but also supports balanced immune regulation. The integrity of the amniotic epithelial barrier is crucial for maintaining a sterile environment in the uterus and preventing the ascent of pathogens from the vagina into the amniotic sac [52,53]. During intrauterine infection, such as chorioamnionitis, this barrier becomes compromised, leading to increased permeability and facilitating the entry of pathogens into the amniotic cavity [39,40,41,42]. Our study also revealed that co-administration of BD2 with LPS mitigated the LPS-induced reduction in E-cadherin expression. Additionally, BD2 treatment downregulated Snail, a transcription factor that suppresses E-cadherin and promotes epithelial–mesenchymal transition, thereby reinforcing its role in preserving epithelial integrity. This function is critical in chorioamnionitis, where barrier disruption can lead to fetal exposure to pathogens and inflammation.

Finally, we investigated the therapeutic potential of BD2 in preventing preterm birth using an LPS-induced mouse model. Recent advancements in understanding the complex pathophysiology of sPTB shifted the focus of treatment from traditional agents, such as tocolytics and antibiotics, to approaches targeting intrauterine inflammation [54,55,56]. Although some promising agents demonstrated favorable results, few new medications have yet been introduced to the commercial market. In this study, preterm delivery occurred within 24 h in the LPS-treated group. In contrast, BD2 administration, both subcutaneously before LPS injection and intrauterinely at the time of LPS injection, significantly delayed delivery, maintaining timing comparable to that observed in control mice. When BD2 was given only subcutaneously, it had little effect in preventing preterm birth. However, intrauterine BD2 administration alongside LPS, without prior subcutaneous treatment, provided some protective effect, though it was less effective than the combination of both subcutaneous and intrauterine BD2 administration (Supplementary Figure S3). These findings suggest that BD2’s protective effect against PTB depends on the route of administration, with local delivery being more effective. The most pronounced effect was observed with both subcutaneous pre-treatment and intrauterine BD2 administration, suggesting that systemic priming may enhance its local activity. However, due to the limited sample size, further validation is needed to draw definitive conclusions. To clarify whether BD2’s effects result from its biological activity or the injection procedure itself, an additional control group, such as an LPS + PBS subcutaneous group, would help address potential confounding factors. Furthermore, BD2 treatment significantly attenuated the LPS-induced elevations in IL-6 and IL-1β levels, suggesting its significant anti-inflammatory properties in vivo. Our results are consistent with previous in vitro findings, where BD2 demonstrated the ability to downregulate pro-inflammatory cytokine expression in hAECs exposed to LPS. The delayed delivery observed in the BD2-treated groups aligns with the cytokine profile, further supporting the hypothesis that BD2 exerts its protective effects by counteracting the inflammatory cascades associated with PTB. Notably, BD2 treatment did not adversely affect normal gestational progression in the absence of LPS. This finding highlights its safety profile and potential for therapeutic application.

The strength of this study lies in its detailed analysis of the role of BD2 in both in vitro and in vivo experimental settings. We conducted experiments using the amniotic membranes of preterm patients diagnosed with chorioamnionitis, demonstrating the changes in BD2 expression in these membranes and highlighting the role of BD2 in the context of chorioamnionitis. To identify the stimuli responsible for changes in BD2 expression, we used LPS as an inflammatory stimulus, showing that BD2 expression is induced by infection/inflammation. Furthermore, by replicating the experimental results obtained from patient-derived epithelial cells isolated from the amniotic membrane, we identified these epithelial cells as a key source of BD2 expression and release. Our findings underscore the significant role of amniotic epithelial cells in regulating the inflammatory response and protecting the epithelial barrier during inflammation-induced PTB. We further demonstrated that BD2 prevented sPTB and significantly extended the gestational period in an LPS-induced PTB mouse model, highlighting its potential as a therapeutic agent. However, this study has several limitations. First, the experiments were conducted using amniotic membranes obtained from a total of 26 patients, which may limit the ability to draw definitive conclusions. Future studies with larger cohorts are needed to examine variations in BD2 levels according to different clinical presentations and disease severity, as well as its potential associations with pregnancy outcomes. Additionally, this study did not address various other actions of defensins, such as their roles in chemotaxis and the activation of diverse immune cells, including neutrophils and macrophages. While BD2 expression was shown to be regulated by NF-κB, previous research indicated that it is also influenced by AP-1 and MAPK pathways and controlled by a complex network of transcription factors [57,58]. However, our study did not explore these molecular interactions or immune signaling pathways in depth. Further research is necessary to elucidate the role of BD2 in PTB by investigating its interactions with various immune signaling pathways and their contributions to host defense mechanisms during pregnancy. Defensins are known to interact synergistically with other AMPs to modulate responses to inflammatory stimuli. Since this study focused exclusively on the isolated effects of BD2, the observed responses might differ from those occurring within the intricate interplay of factors in vivo. Furthermore, the in vivo results primarily highlight short-term outcomes, such as delivery timing and cytokine levels following BD2 treatment. To fully understand its therapeutic potential, long-term studies are necessary to evaluate both the benefits and risks of BD2 treatment in preventing preterm birth or managing chorioamnionitis. For the clinical application of BD2, further investigation is required to address practical challenges, including safety, optimal dosage, and delivery methods. Additionally, potential off-target effects and interactions with other endogenous antimicrobial peptides must be carefully considered to ensure its safe and effective therapeutic use.

## 4. Materials and Methods

### 4.1. Amnion Collection and Processing

Placental samples (*n* = 6) were collected from women diagnosed with chorioamnionitis due to prolonged latency after PPROM and/or preterm labor (median gestational age of 29.3, range 23.3–34.5), with four delivering via cesarean section and the remaining two via vaginal delivery. Additionally, samples were obtained from healthy women undergoing elective cesarean delivery at term in the absence of labor (*n* = 20; median gestational age 38.3 weeks, range 37.5–39.4). The amniotic membrane was manually separated from the chorion and sectioned into small pieces (approximately 1.0 cm^3^ each). Full-thickness placental biopsies were then collected and fixed in formalin. A placental pathologist, blinded to the clinical and laboratory data, analyzed the specimens. Amnion samples histologically diagnosed with acute chorioamnionitis were utilized as patient samples in the experiments. The collection and use of the samples were approved by the Ethics Committee of Kangnam Sacred Heart Hospital (Ethics ref. 2021-05-003).

### 4.2. Immunohistochemistry

Amniotic tissue obtained through cesarean section was fixed in 4% formaldehyde and embedded in paraffin to create blocks. The paraffin blocks were sectioned into 8 μm slices for immunohistochemistry. The sections were deparaffinized with xylene, rehydrated with ethanol, and blocked for 1 h at room temperature with 5% bovine serum albumin. The primary antibody was incubated overnight at 4 °C, followed by a 30 min incubation with the secondary antibody at room temperature. Detection was performed using horseradish peroxidase, and cross-sectional analysis was performed using an Axio Observer optical microscope (Carl Zeiss, Jena, Germany) equipped with a digital camera.

### 4.3. Isolation of Primary Human Amniotic Epithelial Cells

The primary human amniotic epithelial cells were isolated from freshly obtained amnion as described above. The amniotic membrane was washed with ice-cold PBS (pH 7.2) to remove blood clots and cellular debris and cut into 6 cm-long pieces. The first enzymatic digestion was performed by incubating the membrane pieces with 20 mL of pre-warmed 0.05% trypsin/EDTA (Thermo Fisher Scientific, Waltham, MA, USA) at 37 °C for 10 min with gentle shaking. The cells obtained in this step were discarded to remove blood clots and cellular debris. The second and third digestions were conducted in a similar manner. The membrane pieces were transferred into new 50 mL conical tubes containing 20 mL 0.05% trypsin/EDTA and incubated at 37 °C for 30 min with gentle shaking. The digestion mixtures obtained from the second and third digestions were filtered through a 70 μm cell strainer and centrifuged at 500× *g* for 10 min. Cell pellets were suspended and grown in DMEM/F12 (Gibco, Franklin Lakes, NJ, USA) supplemented with 10% fetal bovine serum, 100 U/mL penicillin, 100 μg/mL streptomycin (Sigma-Aldrich, St. Louis, MO, USA), and 10 ng/mL epidermal growth factor (R&D Systems, Madison, WI, USA).

### 4.4. Reagents

Lipopolysaccharides from *Escherichia coli* O55:B5 was purchased from Sigma-Aldrich (St. Louis, MO, USA). Recombinant Human Beta-Defensin 2 was purchased from ProSpec Bio (Hamada, Japan). The transfection reagent was purchased from PolyPlus-Transfection (Strasbourg, France). Antibodies, including beta-actin, anti-human Snail, and anti-human E-Cadherin, were purchased from Cell Signaling, while anti-human Beta-Defensin2 from Abcam (Cambridge, UK). BD2-wild type and BD2-mutant vectors were purchased from VectorBuilder (Chicago, IL, USA). The ONE-Glo Luciferase Assay System was purchased from Promega (Madison, WI, USA).

### 4.5. Western Blot Analysis

Western blot analyses were performed using standard methods. Proteins were separated using 15% and 10% SDS-PAGE and transferred unto a methanol-activated polyvinylidene difluoride membrane. The target protein was detected by attaching the corresponding antibody, and enhanced chemiluminescence was employed for the process.

### 4.6. RNA Isolation and Quantitative PCR (qPCR)

Total RNA from hAEC was isolated using TRIzol reagent (Qiagen, Hilden, Germany). RNA (1 μg) was used to synthesize cDNA using a Maxime RT PreMix Kit (iNtRON Biotechnology, Washington, DC, USA). PCR was performed on Rotor-Gene Q (Qiagen) using Power SYBR Green PCR Master Mix (Thermo Fisher Scientific, Waltham, MA, USA). Primers used were Actin (forward): 5′-GTGCTATCCCTGTACCCCTC-3,’ Actin (reverse): 5′-GGCCATCTCTTGCTCGAAGT-3,’ IL-6 (forward): 5′-AATTCGGTACATCCTCGACGG-3,’ IL-6 (reverse): 5′-GGTTGTTTTCTGCCAGTGCC-3,’ IL-10 (forward): 5′-TCAAGGCGCATGTGAACTCC-3,’ IL-1β (forward): 5′-ATGATGGCTTATTACAGTGGCAA-3,’ and IL-1β (reverse): 5′-GTCGGAGATTCGTAGCTGGA-3.’ Data were analyzed using the 2^−ΔΔCt^ method.

### 4.7. Enzyme-Linked Immunosorbent Assay (ELISA)

The levels of BD2, IL-6, IL-1β, IL-10, IL-8, IL-17, MCP-1, MMP9, MMP8, and MMP2 in the culture media of hACEs were determined using ELISA kits (Cusabio, Wuhan, China) according to the manufacturer’s instructions. The levels of IL-6 and IL-1β in mouse plasma were quantified using ELISA kits (Cusabio, Wuhan, China) according to the manufacturer’s instructions.

### 4.8. Transfection and Luciferase Assay

hAECs were seeded in 6-well plates, and luciferase expression vectors (wild-type and mutant) were transfected using the PolyPlus-Transfection kit. Transfection was performed for 24 h, followed by treatment with LPS for 6 h. Cells were lysed using the ONE-Glo™ Luciferase Assay System, and luminescence and EGFP fluorescence were measured. Promoter activity was relative to that of the wild-type BD2 vector.

### 4.9. Experimental Animals

Pregnant C57BL/6 mice at embryonic day 12 (E12) were purchased from Dooyeol Biotech (Seoul, Republic of Korea) to establish infection-induced preterm birth mouse models using LPS. The research protocol was approved by the Institutional Animal Care and Use Committee (IACUC) of Hallym University (permit number R3 (2021-87)), and all experiments were conducted in accordance with the Guide for the Care and Use of Laboratory Animals by the National Institute of Health.

To better mimic the physiological conditions of inflammation-induced preterm birth, we established a preterm birth model using intrauterine LPS administration. A dose–response curve for LPS was performed, and a dose of 5 μg was determined to be optimal, yielding reliable PTB rates with minimal maternal morbidity and mortality while preserving fetal survival. At this dose, intrauterine injection of LPS resulted in preterm delivery in over 75% of cases within 24 h post-injection.

A total of 41 mice were included in the study and divided into four groups: a control group treated with PBS (*n* = 9), an LPS-treated group (*n* = 8), a BD2-only-treated group (*n* = 11)*,* and a group treated with both LPS and BD2 (*n* = 13). The experimental procedure was as follows: On gestation day 15 (E15), dams received a subcutaneous injection of morphine (5 mg/kg) for analgesia prior to a mini laparotomy performed under isoflurane anesthesia (5%) isoflurane in oxygen at a flow rate of 1.5 L/min. The uterine horns were exteriorized and kept moist using sterile PBS applied with a sterile swab. LPS (2.5 μg per uterine horn, for a total of 5 μg) in 100 μL sterile PBS, or 100 μL sterile PBS alone for controls, was injected into the distal regions of the bilateral uterine horns between the first and second uppermost fetuses, avoiding the amniotic cavities. Fetal viability was confirmed before returning the uterine horns to the abdominal cavity. Post-procedure, the mice were allowed to recover in a heated recovery chamber and were subsequently housed individually. Regular monitoring was conducted until euthanasia. Labor time was calculated as the interval from injection to the delivery of one or more fetuses. Blood samples were collected via retro-orbital bleeding at 6 h post-LPS injection and centrifuged immediately at 3000 g for 15 min, after which serum was separated and stored at −80 °C for subsequent analysis.

### 4.10. Statistical Analyses

All experiments were conducted at least three times independently. Statistical analyses were performed using GraphPad Prism version 8.0 (GraphPad Software, www.graphpad.com, accessed on 1 March 2024). Data were compared using the Mann–Whitney U test or Kruskal–Wallis test, with Bonferroni corrections applied for multiple comparisons. The proportions of sPTB occurrences were compared using Fisher’s exact test. Statistical analysis of immunoassay data was performed after logarithmic transformation. A *p*-value of less than 0.05 was considered statistically significant.

## 5. Conclusions

Our study demonstrates that BD2 plays a crucial role in regulating the inflammatory response and maintaining epithelial barrier integrity in infection/inflammation conditions during pregnancy, such as chorioamnionitis, ultimately contributing to the prevention of sPTB. Further research is needed to explore the precise mechanisms underlying BD2’s protective effects and to evaluate its clinical applicability in prophylactic and therapeutic tools against sPTB.

## Data Availability

The data presented in this study are available on request from the corresponding author.

## Supplementary Materials

The following supporting information can be downloaded at: https://www.mdpi.com/article/10.3390/ijms26052127/s1.
