# Supplementary figures and images for: The Role of Beta-Defensin 2 in Preventing Preterm Birth with Chorioamnionitis: Insights into Inflammatory Responses and Epithelial Barrier Protection
Source: Int J Mol Sci. 2025 Feb 27;26(5):2127. doi: 10.3390/ijms26052127 (PMC11900102; doi:10.3390/ijms26052127)

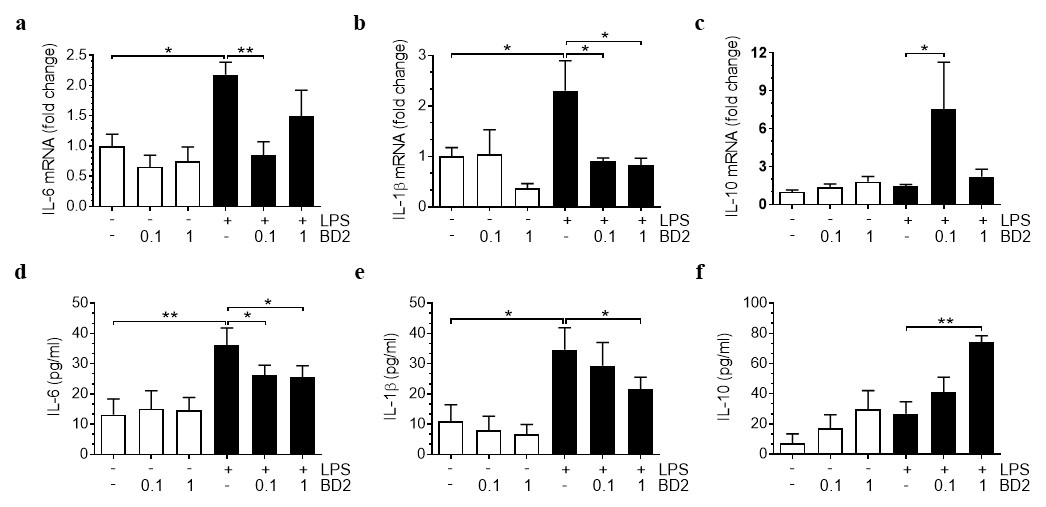

Supplement: Supplementary file 1 [file ijms-26-02127-s001.zip › Supplementary Figure S1.jpg]

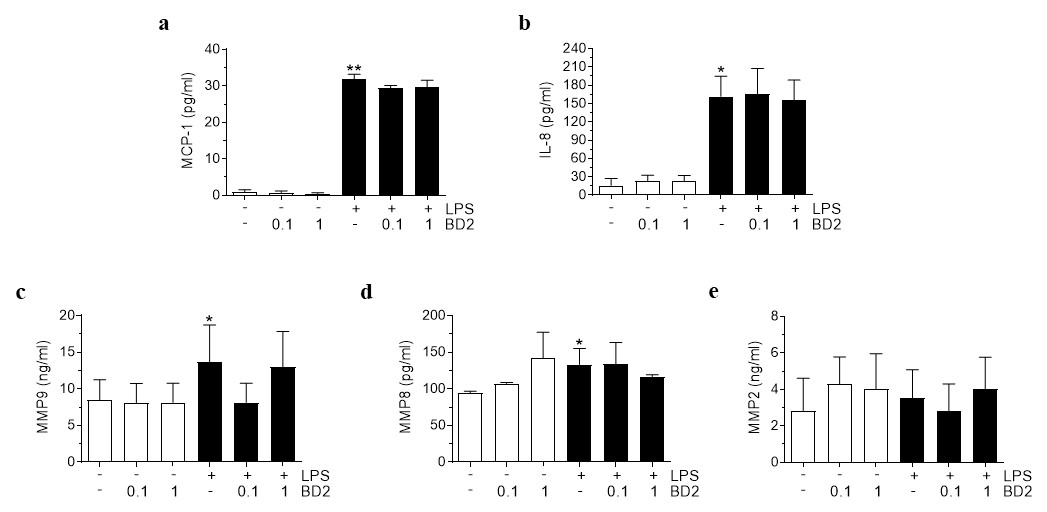

Supplement: Supplementary file 1 [file ijms-26-02127-s001.zip › Supplementary Figure S2.jpg]

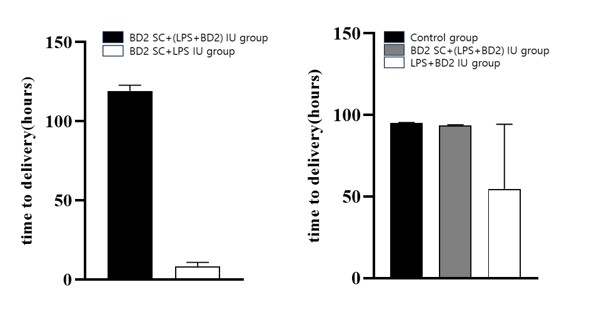

Supplement: Supplementary file 1 [file ijms-26-02127-s001.zip › Supplementary Figure S3.jpg]
